# Bispecific Targeting of EGFR and Urokinase Receptor (uPAR) Using Ligand-Targeted Toxins in Solid Tumors

**DOI:** 10.3390/biom10060956

**Published:** 2020-06-25

**Authors:** Felix Oh, Jaime F. Modiano, Veronika Bachanova, Daniel A. Vallera

**Affiliations:** 1 School of Medicine, Department of Radiation Oncology, University of Minnesota, Minneapolis, MN 55455 USA; ohxxx408@umn.edu; 2 Masonic Cancer Center, University of Minnesota, Minneapolis, MN 55455, USA; modiano@umn.edu (J.F.M.); bach0173@umn.edu (V.B.); 3 College of Veterinary Medicine, Department of Veterinary Clinical Sciences, University of Minnesota, St Paul, MN 55108, USA; 4 School of Medicine, Department of Medicine, Division of Hematology, Oncology and Transplantation, University of Minnesota, Minneapolis, MN 55455, USA

**Keywords:** EGFR (epidermal growth factor receptor), uPAR (urokinase-type plasminogen activator receptor), bispecific, angiogenesis, immunotoxin, LTT (ligand-targeted toxin), KDEL

## Abstract

Ligand-targeted toxins (LTTs) are bioengineered molecules which are composed of a targeting component linked to a toxin that induces cell death once the LTT binds its target. Bispecific targeting allows for the simultaneous targeting of two receptors. In this review, we mostly focus on the epidermal growth factor receptor (EGFR) as a target. We discuss the development and testing of a bispecific LTT targeting EGFR and urokinase-type plasminogen activator receptor (uPAR) as two attractive targets implicated in tumor growth and in the regulation of the tumor microvasculature in solid tumors. In vitro and mouse xenograft studies have shown that EGFR-targeted bispecific angiotoxin (eBAT) is effective against human solid tumors. Canine studies have shown that eBAT is both safe and effective against canine hemangiosarcoma, which is physiologically similar to human angiosarcoma. Finding the appropriate dosing strategy and sequencing of eBAT administration, in combination with other therapeutics, are among important factors for future directions. Together, the data indicate that eBAT targets cancer stem cells, it may have a role in inhibiting human tumor vasculature, and its bispecific conformation may have a role in reducing toxicity in comparative oncologic trials in dogs.

## 1. Targeted Toxins Have Made a Mark on Cancer Therapy

**Figure 1:**
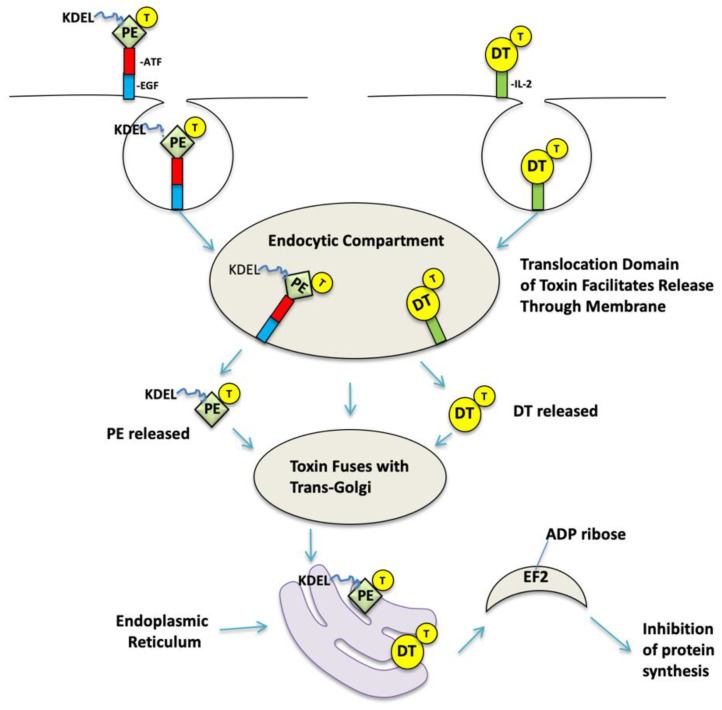
Intoxification Pathway. The figure shows the intoxification of *Pseudomonas aeruginosa* exotoxin A (PE)-based EGFR-targeted bispecific angiotoxin (eBAT) and DTIL2 (Ontak). Regarding eBAT, since the native binding domain has been removed, the molecule binds via epidermal growth factor (EGF) and/or amino-terminal fragment (ATF) to epidermal growth factor receptor (EGFR) and urokinase-type plasminogen activator receptor (uPAR), respectfully. eBAT is internalized in endocytotic vesicles. The toxin contains a natural translocation region (T), translocates the membrane, and is released, where it either traffics to the Golgi or continues to lysosomes for terminal degradation. In the Golgi, PE that is engineered to express C-terminal KDEL encounters KDEL receptors, giving cause to transport PE to the endoplasmic reticulum in a retrograde manner. The toxin fragment containing the catalytic domain is subsequently transported into the cytosol, where it catalyzes the ADP-ribosylation of elongation-2 factor, a vital component of protein synthesis. This is a catastrophic ribosomal event that results in cellular apoptotic cell death. DTIL-2 follows a very similar mechanism of action but is internalized in acidified vesicles.

Immunotoxins are mostly defined as bioengineered hybrid molecules consisting of an immune system ligand (usually antibody fragment), linked to a potent toxin, designed to specifically kill designated target cells [1]. In this review, we discuss a strategy that uses human cytokine ligands linked to toxins to take advantage of high affinity cytokine/receptor interactions and aberrant patterns of the expression of cytokine receptors in cancer cells. These ligand-targeted toxins (LTTs) bind tumors and/or components of their tumor microenvironment and induce toxin uptake. Bacterial toxins that have been used successfully for this purpose include *Pseudomonas aeruginosa* exotoxin A (PE) or diphtheria toxin (DT), both of which have similar mechanisms of cytotoxicity [2,3]. The catalytic domains of PE and DT catalyze the ADP-ribosylation of eukaryotic elongation factor-2 (EF2) (Figure 1). This arrests protein synthesis and causes cell death [1]. It has been calculated that as few as 1000 molecules of bacterial toxin per cell are needed for complete tumor killing, which is achievable at picomolar concentrations of bispecific LTTs [4,5]. LTTs are advantageous for killing tumor cells because of their specificity, cytotoxicity, and their capacity to target multiple cell-surface receptors. One disadvantage of LTTs is that their toxin components are highly immunogenic because of their bacterial origin. In some patients receiving PE-based LTTs, PE induced the development of neutralizing antibodies within 3 weeks of administration [6]. Advances in epitope mapping have reduced the immunogenicity of bacterial toxins, increasing the potential of LTTs for clinical use [7].

Monospecific immunotoxins have successfully reached the market. Though currently discontinued, denileukin diftitox (Ontak) received FDA approval for the treatment of cutaneous T cell lymphoma in 1999 [8]. Ontak was a recombinant molecule composed of truncated DT fused with interleukin 2 (IL-2), a cytokine produced by T lymphocytes. Studies showed that Ontak was efficacious against T cell lymphomas expressing human IL2 receptor CD25 [8]. Tagraxofusp (Elzonris) is a more recent example of a truncated DT immunotoxin fused with human interleukin 3 (IL-3) which received FDA approval for the treatment of blastic plasmacytoid dendritic cell neoplasm (BPDCN) in 2018 [9]. Moxetumomab pasudotox (Moxe) is an anti-CD22 immunotoxin that uses truncated *Pseudomonas* exotoxin as a toxin. Moxe is a chimeric molecule composed of a single-chain fragment variable (scFv) antibody moiety, which recognizes the CD22 receptor on B cells, fused with truncated PE, which triggers cell death [10]. Moxe was evaluated in phase III trials in 2013 and approved by the FDA in 2018 for the treatment of refractory hairy cell leukemia [11].

## 2. Bispecificity Can Be Advantageous over Monospecific Therapy

The binding moiety of a targeted toxin binds to determinants expressed on cancer cells and/or the tumor microenvironment and is largely responsible for its specificity. Investigators have reported certain disadvantages in selecting ligands or antibodies that bind a single target determinant. For example, targeting CD19 in B cell malignancies with CD19-directed blinatumomab resulted in the downregulation of CD19 in clinical studies [12]. A potential solution to this problem is to simultaneously target two different determinants on the cancer target to enhance antigenic recognition and binding. For example, DT2219 is a bispecific immunotoxin that combines the catalytically active domain of a truncated DT (similar to the one used in Ontak) with antibody fragments targeting human CD19 and CD22, both of which are expressed in B cell malignancies [13]. DT2219 is notable since it was among the few bispecifically targeted toxins to reach clinical trials. Phase I trials established a safe and biologically effective dose of DT2219 to be eight doses of 60 μg/kg/day [14]. Phase II determined that patients experienced minimal adverse effects after day 29 of treatment. The most common adverse effects were capillary leak syndrome, increased liver function, and leukopenia that all resolved after 3–5 days and allowed for continued treatment. Of the eighteen patients enrolled in the phase I/II study with CD19 or CD22 positive lymphoma or leukemia, one patient with primary refractory pre-B acute lymphoblastic leukemia achieved complete tumor response after one cycle and two patients with lymphoma experienced partial responses. Further development has focused on reducing the immune response by combining DT2219 with rituximab to deplete B cells and reduce neutralizing antibody formation [15]. DT2219 proved it has greater cytotoxicity against patient leukemia cells than monomeric LTTs made exclusively with anti-CD19 or anti-CD22 antibodies [13,16]. Bispecific agents can simultaneously target two different receptors on the tumor surface to deliver the bacterial toxin payload that allows them to serve a wider range of anti-cancer applications and perhaps overcome the problem of resistance. Bispecific agents are currently undergoing further investigation.

## 3. EGFR, A Prominent Target in Cancer Therapy

Most successful LTT clinical applications relate to targeting liquid tumors. A sizable challenge will be successfully targeting solid tumors. Tumor antigens can be highly evasive due to modulation or shedding, and solid tumors have a highly developed vasculature that promotes growth. In this instance, bispecific targeting may be even more important. EGFR and ErbB-2 are prominent cancer markers. In early studies, investigators developed bispecific LTTs to target solid tumors [17]. scFv2(FRP5/225)-ETA is a bispecific LTT, composed of two scFv domains that target EGFR and ErbB-2 linked to modified PE, which demonstrated more potent anti-cancer activity in vitro and in vivo than its monospecific counterparts, scFv(FRP5)-ETA and scFv(225)-ETA. These studies suggested that bispecific LTTs that target two distinct receptors expressed on the same subset of tumor cells can both overcome deficiencies in individual cell receptor expression and improve the binding specificity of the LTT. We took advantage of the bispecific property of LTTs to engineer a new recombinant drug against solid tumors and focused on a different approach that we proposed would simultaneously target both the tumor with EGF and the tumor vasculature with a high-affinity fragment of urokinase.

Epidermal growth factor (EGFR) is a well-established marker that serves as a valuable target for antibodies, EGFR inhibitors, and targeted toxins. EGFR targeting is advantageous because it is a viable target for multiple tumors with diverse histological and developmental origins, including carcinomas, sarcomas, and brain tumors [18]. EGFR inhibitors are one form of EGFR targeting that blocks the abnormal receptor or signaling pathway to manage solid tumors. For example, mouse monoclonal antibody (MAb) 225 and its derivatives were first found to block EGF binding to EGFR and promote the internalization of the receptor [19]. Cetuximab is a partially humanized MAb225 antibody, with a much higher affinity for EGFR than its parental antibody, that has been approved for the treatment of metastatic colon cancer and squamous cell carcinoma [20]. Panitumumab is a fully humanized MAb225 approved for the treatment of colorectal adenocarcinoma [21]. These inhibitors bind EGFR to prevent its phosphorylation, inhibiting ligand binding and the subsequent activation of tumorigenic kinases [22]. In contrast, LTTs that use EGF as a targeting moiety avoid the tumor cell activation pathway entirely because they ultimately use EGFR to kill the target cell. Early EGFR-targeting LTTs consisting of the scFv of MAb225 fused with PE were found to be potent against breast and epidermal cancers [23]. 

Though less toxic than conventional chemotherapies, EGFR-targeting therapeutics still have notable adverse effects. The ubiquity of EGFR expression in both tumors and healthy tissue renders off-target toxicity an issue with anti-EGFR therapeutics. Though EGFR can be expressed in mutant form or overexpressed in tumors of the mammary glands, lungs, pancreas, intestines, and central nervous system, EGFR can also be found in both healthy tissue and tumors in the wild type form at normal levels [24]. Moreover, EGFR is highly expressed on the skin epithelium, hair follicles, and sebaceous glands, and its inhibition can result in adverse skin reactions [25]. EGFR inhibitors have also been known to cause gastrointestinal, pulmonary, cardiac, ocular, oral, and liver toxicity [25]. Despite the effectiveness of EGFR-targeting drugs, their toxicity narrows the therapeutic index. Attempts have been made to reduce the dose-limiting toxicity of EGFR-targeted drugs by combining EGFR inhibitors with antiangiogenic therapeutics [25,26]. 

## 4. In Addition to Targeting Cancer Cells, Targeted Toxins Can Target the Tumor Microenvironment

Solid tumors require new blood vessels to grow [27]. Vascular endothelial growth factor receptor (VEGFR) is an example of a vascular target that plays a signaling role in angiogenesis, the formation of tumor neovasculature. In healthy tissue, VEGFR binds vascular endothelial growth factor (VEGF) in fetal organs, healing wounds, and under hypoxic conditions [28,29]. Cancerous cells express VEGFR to recruit new blood vessels and meet the increased nutrient demands of solid tumors through the neovasculature. Bevacizumab is an anti-VEGF antibody that blocks the interaction between VEGF and VEGFR. This leads to blood vessel normalization by blocking the recruitment of blood vessel forming cells that stimulate sprouting and branching from the existing vasculature [25]. Historically, the success of anti-VEGFR therapies has made targeted toxins that can target the tumor microenvironment increasingly attractive.

Urokinase plasminogen activating receptor (uPAR) is a glycosylphosphatidylinositol (GPI)-linked receptor that binds urokinase in order to induce the proteolytic reorganization of tissue via phospholipase-C. uPAR is a key receptor involved in cancer signaling pathways that promotes tumor growth and metastasis [30]. Through these pathways, uPAR regulates cell migration, adhesion, and proliferation, especially in cultured endothelial vascular cells [31]. uPAR is a critical vascular target, similar to VEGFR, because of its expression in both cultured endothelial vascular cells and tumor cells, which allows the targeting of existing tumor microenvironments and the inhibition of further angiogenesis and tumor growth [32]. This has been supported by observations that uPAR-directed toxins are effective against human umbilical vein endothelial cells (HUVECs), while EGFR-directed toxins are not [33]. We used a fragment of the urokinase ligand that lacked its catalytic domain but contained its high-affinity binding region, called the amino-terminal fragment (ATF). The cytotoxicity of monospecific ATF LTTs has previously been described in glioma cells in vitro [30,34]. Monospecific ATF LTTs like ATF-KDEL, which is an ATF molecule linked to a modified PE toxin, also raise the potential to target tumor-associated macrophages (TAMs). Macrophages express moderate to high levels of uPAR in blood, bone marrow, lymph nodes, and liver, and have the same pattern of expression in synovial sarcomas [35]. This is important as the immunosuppressive properties of TAMs inhibit natural killer and T cell activity against tumor cells during tumor progression and treatment [36]. TAMs are thus an attractive target for reducing drug resistance and improving the efficacy of antiangiogenic LTTs.

## 5. eBAT, a Bispecific LTT

**Figure 2:**
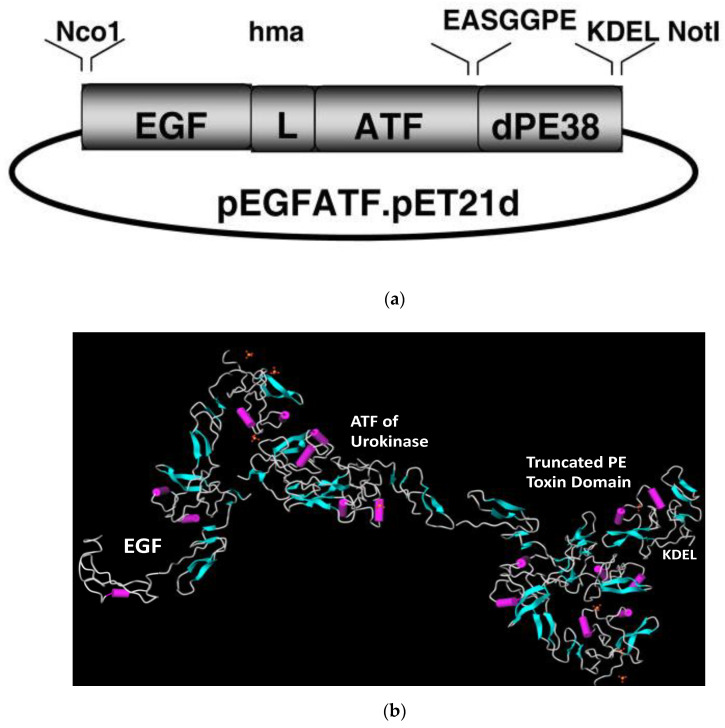
The bispecific LTT eBAT. (**a**) Expression vector for eBAT, human EGF, and the high affinity amino terminal fragment of urokinase linked to a deimmunized PE38KDEL molecule. The fusion gene (from 5′ end to 3′ end) consisted of an NcoI restriction site, the genes for human EGF, an ATG initiation codon, the downstream 135-amino terminal fragment (ATF) from uPA linked by a 20 amino-acid linker (L) of human muscle aldolase (hma), the 7 amino-acid EASGGPE linker, the first 362 amino acids of the pseudomonas exotoxin (PE) molecule with KDEL at the C terminus, and a NotI restriction site at the 3′ end of the construct. (**b**) The ribbon model was assembled using MolView AGPL free software from crystallographic data obtained from the RCSB Protein Data Bank and is not an exact rendering. Accession numbers for crystallographic data are 1IKQ (*Pseudomonas* exotoxin A), 2KV4 (EGF), and 1URK (ATF).

In order to take collective advantage of vascular targeting, EGFR targeting, and simultaneous bispecific targeting, we fashioned a hybrid molecule consisting of human EGF, human ATF, and truncated PE (Figure 2). To enhance its cytotoxic activity, four amino acid residues, Lys-Asp-Glu-Leu (KDEL), were cloned at the C-terminus that enhanced toxin retention in the endoplasmic reticulum (ER) [37]. KDEL is responsible for improving the binding of the LTT to the receptor that transports the toxin to the ER, enhancing the retention of PE and increasing the potency of the toxin [38]. Large foreign toxins have the potential to elicit severe immune responses when administered to humans. Thus, seven major B cell epitopes that provoked neutralizing antibody formation in mice were identified and mutated in the sequence encoding PE [7]. Reducing the immunogenicity of the LTT allowed multiple doses of the drug to be delivered to mice for in vivo use [39]. The LTT was originally called EGFATF-PE and later became known as EGFR-targeted bispecific angiotoxin (eBAT).

## 6. eBAT Studies Show Promise for Glioma and Carcinoma Therapy

In the following sections, we review some of our work detailing how eBAT works against various cancers both in vitro and in vivo. eBAT was first reported by our group as being active against human glioblastoma, a tumor with historically poor treatment prognosis and high EGFR and uPAR expression. eBAT was highly active against human glioblastoma cell lines in vitro, including the U87 and U118 cell lines. Based on the aggressive tumorgenicity of U87 in rat glioma models, U87 was transfected with a luciferase gene for the purpose of real-time bioluminescent imaging [40]. From our in vitro studies, we proceeded to an orthotopic intracranial therapy model. The intracranial treatment effectively reduced brain tumor size in a mouse xenograft model in vivo [41]. In several instances, complete responses were induced. Importantly, eBAT killed U87, an aggressive primary glioblastoma cell line, significantly more effectively than the monospecific LTTs of EGF or the ATF cytokine conjugated to PE38KDEL in vitro. Several courses of treatment with eBAT were associated with a reduction in tumor-associated bioluminescence and longer survival post-treatment compared to the irrelevant negative control and untreated groups [39].

**Figure 3:**
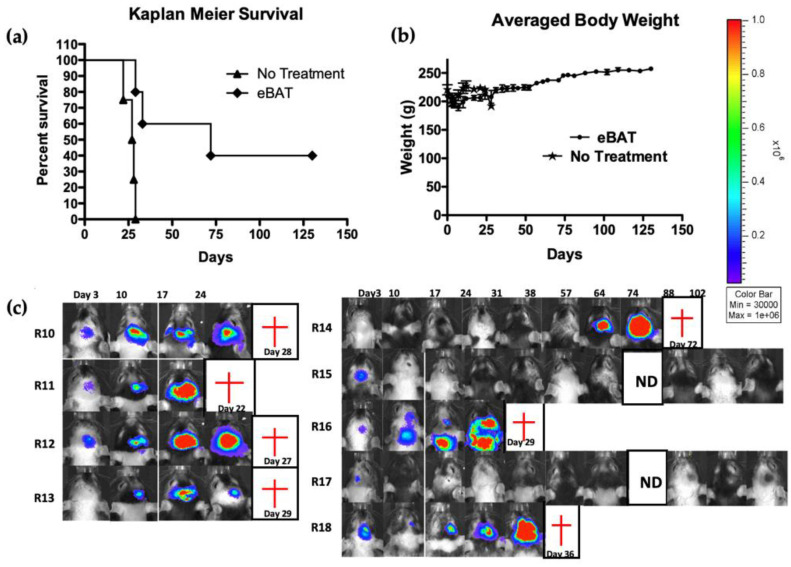
In vivo intracranial tumor experiment showing that eBAT had potent anti-glioma effects [41]. Athymic nude rats were U87-luc cells intracranially on Day 0. On Days 7 and 14, a group of rats was either left untreated or treated with 2 mg eBAT delivered via convection-enhanced delivery (CED), using a hollow fiber (HF) catheter. Drug was delivered stereotactically at the same coordinates as tumor. Each animal was subjected to weekly imaging to monitor tumor growth. (**a**) Kaplan–Meier curve comparing the treated versus the untreated groups. (**b**) Animal Weight. Average weight in grams of animals measured throughout study indicates no long-term weight loss. (**c**) Individual image of each animal in real time. Animals R10 through R13 are not treated and animals R14-R18 are treated with eBAT. Bioluminescence is shown on each picture as a function of photons/s/cm^2^/sr. The intensity of the signal is illustrated by the color bar, with red representing the highest signal intensity. Red crosses indicate death of the animal. ND = no data at that time point. Note that hair patterns vary from week to week because nude rats elicit abortive hair growth. Reprinted with permission of the Journal of Neurosurgery Publishing Group.

U87 proved more tumorigenic in nude rats than in nude mice. Thus, rats were chosen for their increased size, and intracranial tumors were established using U87 cells expressing the luciferase reporter gene (U87-luc). Treatment was administered via a catheter and convection-enhanced delivery (CED). Microinfusion pumps with a hollow fiber (HF) catheter delivered the drug to the same coordinates as the tumor. Treatment resulted in significant tumor reductions in rat brain tumors, with some rats surviving tumor-free for up to 130 days upon the conclusion of treatment (Figure 3). Both in vivo studies established that the LTT could be administered in rodent models without eliciting major immune responses, perhaps because of the “privileged nature” of the brain. Criticisms have been directed at the U87 glioblastoma cell line. It does not grow in the cranium with the same pathophysiology as spontaneously arising glioblastoma and its current genetic profile does not match the genetic profile of its origin [42]. Thus, we chose two other in vivo mouse models.

eBAT was studied in two carcinoma models using the UMSCC-11B human head and neck squamous cell carcinoma (HNSCC) cell line and the triple-negative breast cancer cell line MBA-MB-231 [33,39]. Flow cytometry confirmed that UMSCC-11B and NA-SCC, two chemoresistant head and neck cancer cell lines, were both EGFR positive. In order to confirm the specificity of the ATF component of eBAT to uPAR and prove that eBAT directly reacted with transformed vascular endothelial cells, reactivity was confirmed against uPAR+ HUVECs. UMSCC-11B-luc flank tumors were established in mice and treated intratumorally with eBAT for 4 weeks. Treatment with eBAT inhibited tumor growth [33]. eBAT killed UMSCC-11B tumors in vitro and significantly inhibited tumors in mouse models in vivo, which supported our belief that the bispecificity of eBAT overcame the low expression levels of uPAR in human HNSCC [33].

## 7. eBAT Shows Efficacy in Sarcoma Xenograft Models and Cancer Stem Cells

A recent area of study for eBAT is in human sarcomas, a group of rare and aggressive tumors that originate from the mesenchyme [43]. Consistent expression of EGFR and uPAR (PLAUR mRNA) was found across human sarcomas in the Cancer Genome Atlas (TCGA); similarly, both proteins were found to be expressed in a synovial sarcoma tissue microarray [35]. EGFR targeting has historically translated to poor outcomes in pediatric sarcoma patients and uPAR targeting had not been attempted before [44]. Flow cytometry confirmed the expression of both EGFR and uPAR in the RH30 rhabdomyosarcoma (RMS) cell line and the expression of only uPAR in the TC-71 Ewing sarcoma (EWS) cell line. We then measured the in vitro activity of eBAT against the two sarcoma cell lines using radiolabeled leucine incorporation assays. eBAT inhibited protein synthesis at subnanomolar concentrations in both cell lines, but it was six times more potent in RH30 cells than TC-71 cells. RH30 and TC-71 tumors expressing the luciferase reporter gene were also established in mice and were treated with eBAT. eBAT was found to be effective against pediatric RMS and EWS, both in vitro and in vivo, but the dose that was required to kill the cells that expressed both receptors was an order of magnitude lower than the dose required to kill cells that expressed only uPAR. 

Cancer stem cells (CSCs) represent the progenitor fraction of cancer cells that are chemotherapy and radiation resistant. Tumor spheroid assays are one way of studying CSCs in vitro. Studies by Pilbeam et al. showed that eBAT effectively reduced the number of cells in non-adherent spheroid cancer cell cultures of human RH30 sarcoma cells [44]. This has implications for when and how eBAT should be used and supports its use in combination with chemotherapy. While eBAT targets CSCs, chemotherapy kills the transit-amplifying cells, which are undifferentiated cells arising from stem cells that differentiate abnormally and accumulate into cancer [45].

Recently, eBAT was found to be effective against adult rhabdomyosarcoma and human osteosarcoma in vitro [46]. Flow cytometry confirmed the specific binding of fluorescently labeled eBAT to EGFR and uPAR expressing RD, a rhabdomyosarcoma cell line, and Saos2, an osteosarcoma cell line. We then used both thymidine and leucine incorporation assays to test the cytotoxicity of eBAT against RD and Saos2 cells in vitro. eBAT killed both the rhabdomyosarcoma and the osteosarcoma cells more effectively than its monospecific counterparts, EGF-KDEL and ATF-KDEL, and it demonstrated significantly greater potency than its monospecific counterparts in the two cell lines.

## 8. eBAT Is Effective in Canine Companion Clinical Trials

Having established its efficacy in small animal rodent models, we sought to validate the drug’s usefulness in large animals. We investigated the hypothesis of whether or not bispecific targeting would benefit the efficacy of targeted toxins against hemangiosarcoma (HSA). HSA was chosen for a variety of reasons. HSA is an aggressive and incurable cancer derived from blood vessel forming cells that are highly resistant to chemotherapy and are host to a subpopulation of CSCs that further amplify drug resistance. The co-expression of EGFR and uPAR by HSA cells supported the clinical use of eBAT. Through in vitro studies, we observed that eBAT bound and killed canine tumor cells at picomolar concentrations [47]. In the study, canine HSAs were allowed to develop into non-adherent tumor spheres (hemangiospheres), which are enriched with chemoresistant CSCs, and treated with eBAT. The results showed that eBAT was also effective against canine hemangiospheres in vitro at clinically relevant nanomolar to picomolar concentrations.

**Figure 4:**
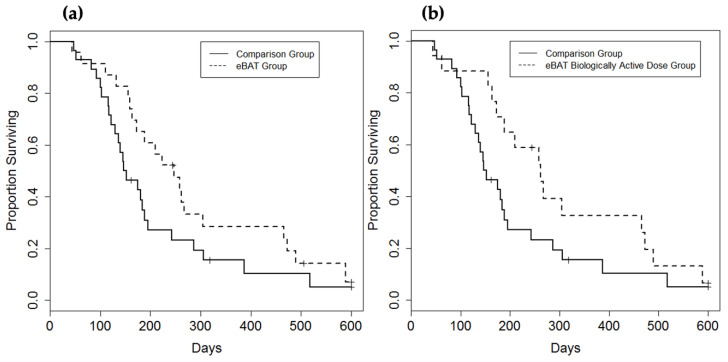
Effect of eBAT on survival of dogs with splenic hemangiosarcoma treated with adjuvant doxorubicin chemotherapy [35]. (**a**) Kaplan–Meier curve for all 23 dogs in the SRCBST-1 study versus the comparison dogs which received standard of care treatment. (**b**) Kaplan–Meier curve for the 17 dogs treated at the biologically effective dose versus the comparison dogs. Curves illustrate prolongation of survival in dogs treated with eBAT compared with the comparison group. Reprinted with permission of American Association for Cancer Research Journals.

Canine sarcomas can be useful models for human sarcomas [48]. The eBAT studies in large animals were completed through the University of Minnesota Veterinary School, which has a canine oncology clinic that specializes in treating companion dogs. A clinical study was undertaken with twenty-three domestic dogs with spontaneous HSA that underwent splenectomy and were treated intravenously with eBAT in advance of five cycles of doxorubicin. This adaptive dose-finding study established a biologically effective dose that was both safe and effective in dogs [49]. eBAT was well tolerated at the biologically effective dose of 50 μg/kg and significantly improved the median survival time when compared to dogs which were treated with the standard of care alone (Figure 4). Modest liver toxicity, inferred from elevated liver enzymes, was self-limiting and reversible. None of the dogs experienced other acute or chronic toxicities associated with eBAT therapy.

For the first time, eBAT was found to be effective in non-xenografted large animal sarcomas in a study design similar to a human clinical trial [35,47]. eBAT was conducive to long-term survival as the proportion of dogs receiving the biologically effective dose that survived for 6 months was almost double that of the comparison group, with six dogs surviving over 450 days [35]. eBAT was also well-tolerated, supporting our hypothesis that bispecificity reduces the toxicity associated with EGFR-targeting. Interestingly, chemotherapy was delayed and associated with prolonged survival in dogs in the treatment group relative to the comparison group [35]. A shorter time between the administration of multiple cycles of eBAT and the initiation of doxorubicin chemotherapy increased adverse outcomes without significantly prolonging survival [50]. This elucidates a possible mechanism of action whereby adding chemotherapy too close to eBAT diminishes or eliminates their efficacy, possibly due to the generation of an immune response. One possible interpretation is that the immune response and the development of a neutralizing antibody following eBAT dampens its efficacy, and chemotherapy may eliminate immune cells and augment potency. We learned that eBAT is unlikely to be an effective anti-cancer therapy unless it is combined with other therapies and timed correctly. The canine trials generated valuable information that will be used to launch a future combination-therapy human phase I clinical trial.

## 9. Bispecific Targeting Appears to Reduce eBAT Toxicity, a Major Drug Advantage

An important finding related to the canine eBAT studies is the reduced toxicity attributed to the addition of the uPAR targeting ligand to the bispecific molecule. Interestingly, in the clinical canine trials, none of the eBAT-treated dogs experienced signs of capillary leak syndrome, the toxicity which is of the greatest concern for LTTs [15,51]. Furthermore, there was a lack of adverse events typically caused by EGFR-targeted therapies. This suggests that it may have been the bispecificity and the addition of the uPAR-directed ligand that enhanced tumor targeting and diminished toxicity. However, the data should be interpreted cautiously, as the specific mechanisms that mediate this reduced toxicity remain to be elucidated and could be dependent on important physiological differences between humans and dogs.

## 10. Discussion

Few LTTs delivering bacterial toxin payloads have made it to FDA-approved status. The road to approval is difficult and there are many barriers that eBAT must overcome before it can reach human clinical trials. Though the current in vivo data in small and large animals are promising, the appropriate timing, dosage, and delivery of the treatment in humans remain to be determined. We expect future pharmacokinetic studies of the metabolism of eBAT in dogs to help us optimize the appropriate dosing and schedule of eBAT in human patients. The development of an immunogenic response to the LTT is also a consideration. On the basis of response criteria, physicians may decide that patients are ineligible to repeat cycles of LTT therapy due to the development of antitoxin antibodies after their first cycle. Interestingly, in a recent report, Kreitman et al. reported on *Pseudomonas*-based Moxe in leukemia clinical trials in which anti-drug antibodies were detected in 84% of evaluable patients after two drug treatment cycles [52]. Antibody levels were higher in patients with progressive and stable diseases as compared to patients with remissions. Despite this, patients with anti-drug antibodies were still able to achieve complete remissions, some without minimal residual disease. The investigators concluded that neutralizing antibodies did interfere with efficacy, at least at high levels, but Moxe was still effective despite the significant immunogenicity. 

It is also worth mentioning that other bispecific ErbB targeting agents have already undergone clinical evaluation. LY3164530 is a bispecific antibody that targets mesenchymal-epithelial transition factor (MET) and EGFR for treating advanced and metastatic cancer that recently underwent phase I trials [53]. Though partial responses were achieved in three patients, the high toxicity prevented its phase II development. MM-111 is a bispecific antibody that targets and inhibits ErbB-2 and ErbB-3 signaling in heregulin positive tumors [54]. Though it completed phase I trials for metastatic breast cancer and gastroesophageal cancer, MM-111 was discontinued after phase II because most patients fell below the threshold of heregulin expression that was needed to benefit from the treatment. This is interesting, especially since we do not know if there will be a threshold of expression needed for eBAT safety or efficacy.

## 11. The Future

In conclusion, eBAT has several advantages and disadvantages (Table 1). eBAT has proven to be more effective than its monospecific counterparts in small and large animal studies. Based on in vivo and canine data, eBAT will likely be most effective in combination with chemotherapy and in patients with minimal residual disease [44]. Unlike other bispecific molecules, eBAT simultaneously targets two important receptors on the cell surface that are implicated in tumor proliferation and vascularization and delivers a potent bacterial toxin that can induce tumor killing at picomolar concentrations. In addition to killing cells by inhibiting protein synthesis, eBAT seems to effectively target the tumor propagating cell population. eBAT targets undifferentiated CSCs as well as “bulk” or transit-amplifying tumor cells. eBAT appears to have a unique mechanism of action whereby the ATF component targets the neoangiogenic vasculature by destroying the tumor microenvironment that recruits new blood vessels. The ATF component also allows the potential targeting of immunosuppressive TAMs, which have been implicated in drug resistance [35]. Our canine studies have shown that dose and schedule appear to be key to maximizing the efficacy of eBAT. An adaptive dose-finding study found that the biologically effective dose did not need to reach the maximum tolerated dose in order to be safe and effective [35]. Our observations also indicate that timing the administration of eBAT too close to the initiation of chemotherapy seems to cause adverse effects. eBAT will likely be most effective in combination with other standard of care therapies that synergize with it or reduce neutralizing antibody formation. The modifications that improved the potency and reduced the immunogenicity of the toxin will reduce the likelihood of eBAT developing neutralizing antibodies and improve patient outcomes. Going forward, on the basis of the current in vivo data and clinical data from similar *Pseudomonas* reagents, we believe that the bispecific LTT eBAT has a promising clinical future.

## Figures and Tables

**Table 1:** A summary of the advantages and disadvantages of eBAT, as described in this review.

**Advantages**	-New molecule (first in class dual targeting tumor and its microenvironment)
	-Targets tumor cells at picomolar concentrations
	-Applicable against a wide variety of solid tumor types
	-Exceptionally safe in vivo
	-Targets cancer stem cells (CSCs) and transit amplifying tumor cells
	-Potential targeting of neoangiogenic vasculature and immunosuppressive TAMs
	-Biologically effective dose does not need to reach maximum tolerated dose
**Disadvantages**	-Modest liver toxicity from dog data-appears to be self-limiting and reversible
	-Likely will need to be used in combination with other therapies
	-Still may generate some antibody response
